# Effect of platelet-rich fibrin in enhancing healing after impacted mandibular third molar extractions: A randomized controlled trial

**DOI:** 10.6026/973206300214167

**Published:** 2025-11-15

**Authors:** Richa Bahadur, Anshumali A, Sajjad Salam, Vishal Kulkarni, Navin Kumar D, Vikram Karande, Anukriti Kumari

**Affiliations:** 1 Department of Dentistry, ESIC Medical College & Hospital, Namkum, Ranchi, Jharkhand, India; 2 Department of Anaesthesia, ESIC Medical College & Hospital, Namkum, Ranchi, Jharkhand, India; 3 Dental and Maxillofacial Services, Bahrain Defense Forces - Military Hospital, Bahrain; 4 Department of Oral and Maxillofacial Surgery, Army Dental Centre Research and Referral, Delhi Cantt, India; 5 Department of Periodontology, Madha Dental College, Chennai, Dr. MGR Medical University, Guindy, Chennai, Tamil Nadu, India; 6 Department of Oral & Maxillofacial Surgery, D.Y. Patil Dental School, Lohegaon, Pune, Maharashtra, India; 7 Department of Oral Medicine and Radiology, School of Dental Sciences, Sharda University, Greater Noida, Uttar Pradesh, India

**Keywords:** Platelet-rich fibrin, third molar extraction, bone regeneration, postoperative healing, pain reduction

## Abstract

The effect of platelet-rich fibrin (PRF) in improving healing post-impacted mandibular third molar extractions is of interest. Hence, a randomized trial was conducted with 30 patients, comparing outcomes between those treated with PRF and those without. The results demonstrated that PRF significantly reduced pain and swelling and promoted better bone healing, particularly in the early postoperative period. Although benefits plateaued at 6 months, PRF remains a promising adjunct for enhancing recovery in oral surgery.

## Background:

Wisdom teeth, also known as third molars, are the teeth that erupt last into the oral cavity and have a prevalence of impaction from 33% to 58.7% [1]. These can be associated with regional consequences such as pericoronitis, regional pain, trismus and abscesses of odontogenic origin, distal caries, as well as cysts or tumors and arch crowding. Therefore, symptomatic or asymptomatic impacted third molars are extracted for the prevention of more serious problems in later clinical practice [2]. Among these, the surgical removal of an impacted third molar of the mandible represents a complex operation and post-operative complications are frequently encountered such as pain, swelling, trismus, nerve damage, haemorrhage and alveolar osteitis (AO) which can interrupt healing and reduce the patient's quality of life [3]. Local anaesthesia is essential during the removal of impacted third molar teeth to achieve good pain relief. Injecting a local anesthetic such as Lidocaine, Articaine or Mepivacaine into the tissue around the tooth to block Nerve conduction and Pain sensation is standard practice [4]. Several methods, such as inferior alveolar (for lower molar) nerve blocks and both buccal and lingual infiltrations, are utilized to obtain sufficient analgesia. The inferior alveolar nerve block - the one that numbs the mandible - is generally plan A [5]. Vasoconstrictors such as epinephrine can be used in local anesthetics to increase the duration of anesthesia and discourage bleeding. Although local anesthetics are in general quite satisfactory, some postoperative pain may be present [6]. It has therefore prompted research into adjuvant therapies, like platelet-rich fibrin (PRF), which can improve healing and decrease pain, swelling and complications even more [7]. Growth factors have generated interest as activators that lead to accelerated wound healing through stimulation of cell proliferation, synthesis of collagen and angiogenesis (generation of new blood vessels) [8]. Platelet-rich plasma (PRP), which was developed in 1997, was one of the earliest members of platelet-derived growth factors for promoting oral and maxillofacial healing. PRP has been demonstrated to accelerate the healing process and also suppress inflammation and pain; however, it is confounded by its short degradative period immediately after implantation [9]. PRF is a second-generation platelet concentrate that has been developed more recently. Not like PRP, PRF maintains the sustained release of growth factors with its fibrin matrix that entrap platelets, leukocytes, cytokines and circulating stem cells [10]. This physiologic property makes PRF an ideal biomaterial for augmentation of soft tissue and bone regeneration. PRF has been applied to oral surgery procedures including, but not limited to, sinus lifts, implant placements and socket preservation, which displayed its potential in minimizing the postoperative complications and facilitating bone formation [11]. However, despite the high efficiency of PRF in a variety of oral surgery procedures, its application in mandibular third molar extractions is minimally investigated especially for soft tissue and bone healing [12]. The majority of the studies assessed PRP and other PC and had only few comparative evaluations to PRF. In view of the difference in demographics and surgical variables, especially from northern India, a further look on use of PRF for enhancing post-operative wound healing is warranted [13]. Therefore, it is of interest to determine the efficacy of PRF in improving soft tissue healing and bone regeneration in patients undergoing impacted mandibular third molar extractions.

## Materials and Methods:

This prospective randomized study was conducted in the Oral & Maxillofacial Surgery OPD at BIDSH, Patna, involving patients with impacted mandibular third molars aged over 18 years. The patients were recruited based on their attendance at the OPD, written informed consent and eligibility according to the inclusion criteria. A prospective randomized study was conducted from December 2021 to December 2024. The study included a total of 30 patients, divided equally into two groups, with 15 patients in each group. Group I included patients who received platelet-rich fibrin (PRF) post-extraction, while Group II did not receive PRF. Sample size was calculated based on a clinically significant difference of 15 score, with a standard deviation of 17.4. Using the formula for sample size calculation and assuming equal sample sizes for both groups, the required sample size per group was found to be 15, leading to a total of 30 patients. The inclusion criteria for this study were as follows: patients aged over 18 years with clinically and radiographically confirmed impacted mandibular third molars, recurrent pericoronitis or associated food impaction, presence of proximal caries on the adjacent molar, chronic or recurrent pain in the region and orthodontic referral for removal of impacted third molars. The exclusion criteria included patients less than 18 years, individuals with generalized chronic destructive periodontitis, medically compromised patients who were contraindicated for surgery and those with wisdom teeth associated with mandibular fractures. Before the surgical procedure, all patients underwent a comprehensive clinical and radiological evaluation. This included measuring the maximum mouth opening using a Vernier's caliper to assess the inter-incisal distance. Extraoral and intraoral photographs were taken for documentation and analysis. An Orthopantomogram (OPG) was performed to evaluate the position of the impacted third molar and surrounding structures. Additionally, routine hematological investigations were conducted to ensure the patients were fit for surgery and to assess their overall health status. Blood was drawn from the antecubital region for PRF preparation (for Group I only), followed by centrifugation for 12 minutes at 3000 rpm. Chlorhexidine gluconate mouthwash was given to patients 10 minutes before surgery. After draping the surgical site with povidone-iodine, 2% lignocaine hydrochloride with adrenaline was administered for local anesthesia. A standard Ward's incision was made using a B.P. blade No. 15 and a mucoperiosteal flap was raised. Bone removal was done using a straight fissure bur No. 702. After tooth extraction, bone margins were smoothed and the socket was irrigated with saline. In Group I, PRF was placed in the extraction socket and the site was sutured. In Group II, no PRF was placed and the extraction socket was sutured with a 3-0 silk suture. Primary closure was achieved with a 3/4th circle reverse cutting needle. Ten milliliters of blood was drawn into test tubes without anticoagulants and centrifuged for 12 minutes at 3000 rpm. Three layers were formed: red blood cell layer at the bottom, PRF clot/gel in the middle and serum on top. PRF clots were retrieved and applied to the extraction site, while the red blood cells and serum were discarded. Days 2, 7, 1 month, 3 months and 6 months for pain, swelling, mouth opening, dry socket and wound dehiscence. Pain was evaluated using the visual analog scale (VAS). Swelling was measured based on specific soft tissue points and mouth opening was assessed with a metallic ruler. Dry socket and wound dehiscence were clinically evaluated.

## Results:

Table 1 (see PDF), Figure 1 (see PDF) shows the distribution of patients on the basis of the gender and the majority patients were female (53.3% in cases and 60.0% in controls) followed by males (46.7% in cases and 40.0% in controls) and the association was found to be statistically insignificant (p>0.05). Table 2 (see PDF) shows the distribution of patients on the basis of their age and the majority patients were in the age range 21-30 years (80.0% in cases and 60.0% in controls) and the association was found to be statistically insignificant (p>0.05) and also the association between the mean ages of the cases and controls was insignificant (p>0.05). Table 3 (see PDF), Figure 2 (see PDF) shows the VAS score on second day and the seventh day where the association was statistically significant between the cases and the controls (p<0.05) whereas the association was statistically insignificant after one month follow up (p>0.05). The swelling in PRF group were decrease at time duration of follow-up but in control group it was increases statistically significant in the control group (p<0.05), it was statistically insignificant after 2nd of postoperative follow up (p>0.05). The Mouth opening was statistically higher in the PRF group as compared to the control group (p<0.05) after seventh day, one month and three month follow up while it was statistically insignificant at six month follow up (p>0.05). On radiographic examination bone healing was statistically higher in the PRF group as compared to the control group (p<0.05) after seventh day, one month and three month follow up while it was statistically insignificant at six month follow up (p>0.05) (Table 6 - see PDF). None of the patients showed complications in the PRF group while in control group wound Dehiscence in 5 (33.3%) patients, paresthesia in 2 (13.3%) and alveolar osteitis (dry socket) in 4 (26.7%) patients. The oral hygiene status was statistically higher in the PRF group as compared to the control group (p<0.05) at second and seventh day, follow up while it was statistically insignificant at one month, three month and six month follow up (p>0.05). The comparison of postoperative swelling between the PRF and control groups shows that the swelling was significantly lower in the PRF group on the second day post-operation (p<0.05). However, the difference between the two groups was statistically insignificant on subsequent follow-ups (7th day, 1st month, 3rd month, and 6th month) (p>0.05) (Table 4 - see PDF). The analysis of mouth opening and radiographic bone healing demonstrated that the PRF group had significantly better results at the 7th day, 1st month, and 3rd month follow-ups (p<0.05) compared to the control group. However, at the 6th month follow-up, the differences between the two groups were statistically insignificant (p>0.05) (Table 5 - see PDF, Figure 3 - see PDF). The comparison of wound dehiscence between the PRF and control groups shows no wound dehiscence in the PRF group at any follow-up stage. However, in the control group, 33.3% of patients had wound dehiscence on the second day post-operation, with a gradual decrease in subsequent follow-ups (Table 7 - see PDF). The analysis of paresthesia between the PRF and control groups reveals that none of the patients in the PRF group experienced paresthesia at any stage, while 13.3% of patients in the control group experienced paresthesia on the second day, with a decrease in later follow-ups (Table 8 - see PDF). The occurrence of alveolar osteitis (dry socket) was significantly higher in the control group, with 26.7% of patients affected on the second and seventh days. No patients in the PRF group developed alveolar osteitis at any follow-up stage (Table 9 - see PDF). The oral hygiene status was significantly better in the PRF group compared to the control group, with 93.3% of patients in the PRF group having good oral hygiene on the second and seventh day follow-ups, compared to only 46.7% in the control group (Table 10 - see PDF).

## Discussion:

This study aimed to evaluate the efficacy of platelet-rich fibrin (PRF) in improving soft tissue healing and bone regeneration following impacted mandibular third molar extractions. The findings suggest that PRF significantly reduced pain, swelling and improved mouth opening during the early postoperative period, as well as facilitated faster bone healing. Pain scores (measured using the Visual Analog Scale, VAS) were significantly lower in the PRF group on days 2 and 7 compared to the control group, though no significant difference was observed after one month. Similarly, swelling was reduced in the PRF group at the 2-day mark, whereas no significant difference was noted after 7 days and beyond. These results align with previous studies, including Hajibagheri *et al.* (2025) [14] who reported similar benefits of PRF in oral surgery, particularly in reducing post-surgical pain and swelling. Further, the improved mouth opening observed in the PRF group, especially in the initial days post-surgery, supports the work of Barone *et al.* (2025) [15], which showed that PRF accelerates the recovery of jaw mobility. Additionally, bone healing was significantly improved in the PRF group after 7 days, 1 month and 3 months, similar to the findings of Al-Maawi *et al.* (2021) [16], where PRF promoted better bone regeneration post-surgery. However, these improvements were less significant at the 6-month mark, aligning with the study of Jamjoom *et al.* (2024) [17] and Acerra *et al.* (2025) [18], which observed that while PRF aids initial healing, its benefits tend to plateau over time. In comparison to the control group, the PRF group showed fewer complications, including wound dehiscence, paresthesia and alveolar osteitis (dry socket), reinforcing the findings of Laforgia 2024 *et al.* (2024) [19], which emphasized PRF's role in minimizing postoperative complications. This study's findings are promising, but further research with a larger sample size and more extended follow-up periods would be valuable to confirm the long-term benefits of PRF in mandibular third molar extractions. When compared to the control group, the PRF group exhibited a lower incidence of complications, including wound dehiscence, paresthesia, and alveolar osteitis.. Such advantages suggest that PRF may play a crucial role in reducing the frequency and severity of common post-extraction complications. The study by Zwittnig *et al.* (2024) [20] aimed to assess the effectiveness of platelet-rich fibrin (PRF) in enhancing postoperative outcomes following third molar extractions. Conducted as a randomized controlled split-mouth trial, the research involved 25 patients who underwent bilateral third molar extractions. In the test group, one side was treated with solid PRF clots, while the other side received conventional treatment. The primary outcome measured was swelling, and secondary outcomes included trismus, pus formation, hematoma, and clinical attachment loss (CAL) of the second molars at days 1, 3, 7, and 14 post-surgery. Additionally, patient-reported outcomes and the consumption of painkillers and antibiotics were recorded from days 0 to 7.

In their 2025 study, Aliberti *et al.* (2025) [21] explored the use of injectable platelet-rich fibrin (i-PRF) in improving postoperative outcomes following impacted mandibular third molar extractions. This double-blind, randomized controlled trial involved 56 patients who were assigned to either an i-PRF treatment group or a control group. The study assessed pain, swelling, and wound healing at multiple time points, including days 1, 3, 7, 14, and 21 post-surgery. Results showed that the i-PRF group experienced significantly reduced swelling on days 3 and 7 compared to the control group, with a notable reduction in pain scores on days 1, 4, and 7 post-surgery. Additionally, the Healing Index scores demonstrated improved soft tissue healing in the i-PRF group across all evaluation days. The study concluded that i-PRF effectively reduces postoperative swelling and pain while promoting faster soft tissue healing. These findings suggest that i-PRF could be a beneficial adjunct in oral surgery, offering better postoperative management and recovery for patients undergoing third molar extractions. However, further studies with larger sample sizes and longer follow-up periods are needed to confirm the long-term benefits and potential of i-PRF in clinical practice. Lu *et al.* (2024) [22] conducted a systematic review and meta-analysis in 2024 to assess the role of platelet-rich fibrin (PRF) in postoperative recovery following third molar extractions. The study included a comprehensive analysis of existing clinical trials and studies that evaluated the effects of PRF on various postoperative outcomes, including swelling, pain, and bone healing. The meta-analysis revealed that PRF significantly reduced swelling and pain in the early postoperative period compared to control treatments. Moreover, PRF was associated with improved bone healing outcomes, including faster osseous regeneration and enhanced bone density at the extraction site. The findings indicated that PRF not only accelerates soft tissue recovery but also contributes to better bone healing, making it a valuable tool in post-surgical care. Despite these positive results, the authors acknowledged the need for further well-designed clinical trials with larger sample sizes to explore the long-term effects of PRF and to refine its application protocols. This study adds to the growing body of evidence supporting the efficacy of PRF in enhancing recovery after third molar extractions and suggests that it may be a promising adjunct to conventional surgical techniques. Iftikhar *et al.* (2024) [23] conducted a triple-blind, split-mouth randomized controlled trial involving 70 patients to assess the efficacy of platelet-rich fibrin (PRF) in enhancing both soft and hard tissue healing following impacted mandibular third molar extractions. The study evaluated multiple postoperative parameters, including pain, bleeding, tenderness, and bone height over a 6-month follow-up period. The results showed that the PRF group experienced significant improvements in soft tissue healing, with reduced pain, bleeding, tenderness, and sensitivity at various time points compared to the control group. These outcomes suggest that PRF plays a significant role in promoting soft tissue recovery after oral surgeries. However, no significant differences were observed in bone height between the two groups, indicating that PRF may have a limited effect on hard tissue regeneration in the context of mandibular third molar extractions. The study concluded that PRF offers substantial benefits in soft tissue healing but may have limited impact on bone regeneration in specific surgical contexts. The authors highlighted the need for further research to explore the full potential of PRF, especially in relation to hard tissue healing and its role in different surgical approaches.

## Conclusion:

PRF significantly enhances soft tissue healing and bone regeneration following mandibular third molar extraction. Data shows PRF can reduce postoperative pain, swelling and complications, making it a valuable adjunct in oral and maxillofacial surgeries. Further studies with a larger sample size are needed to solidify these findings.

## References

[1] Salam S (2023). J Pharm Bioallied Sci..

[2] Peñarrocha-Diago M (2021). J Clin Exp Dent..

[3] Pushpendra S (2025). Oral Sphere J Dent Health Sci..

[4] Decloux D, Ouanounou A (2020). Int Dent J..

[5] El-Kholey K.E (2017). J Maxillofac Oral Surg..

[6] Sisk A.L. (1992). Anesth Prog..

[7] Grecu A.F (2019). Medicina (Kaunas)..

[8] Veith A.P (2019). Adv Drug Deliv Rev..

[9] Bahadur R (2024). J Dent Panacea..

[10] Calciolari E (2025). Periodontol 2000..

[11] Ucer C (2023). Dent J (Basel)..

[12] Dar M.M (2018). Ann Maxillofac Surg..

[13] Miron R.J (2021). Clin Oral Investig..

[14] Hajibagheri P (2025). BMC Oral Health..

[15] Barone S (2025). Head Face Med..

[16] Al-Maawi S (2021). Int J Implant Dent..

[17] Jamjoom A.G. (2024). Cureus..

[18] Acerra A (2025). J Clin Med..

[19] Laforgia A (2024). Int J Mol Sci..

[20] Zwittnig K (2024). Clin Oral Investig..

[21] Aliberti A (2025). Clin Oral Investig..

[22] Lu Y (2024). Journal of Cranio-Maxillofacial Surgery..

[23] Iftikhar I (2024). J Dent Res Dent Clin Dent Prospects..

